# Acute Proptosis: A Sign of Venous Hemorrhagic Infarct

**DOI:** 10.7759/cureus.30891

**Published:** 2022-10-31

**Authors:** Waseem M Ilyas, Gajanan Chavan

**Affiliations:** 1 Emergency Medicine, Jawaharlal Nehru Medical College, Wardha, IND

**Keywords:** transverse venous sinus, sigmoid venous sinus, altered sensorium, emergency department, cerebral venous sinus thromboses, venous hemorrhagic infarct, acute proptosis

## Abstract

Acute proptosis is a very rare condition presenting to the emergency department. As there are very few case reports of patients with acute onset proptosis, it is important to report each new case. This case report is of a 38-year-old lady who presented to our emergency department with a headache for three days, altered sensorium for eight hours, and acute proptosis of the left eye for 40 minutes. She was diagnosed to have a venous hemorrhagic infarct in the left parietal-occipital-temporal region with thrombosis of the left transverse and sigmoid sinuses. To the best of our knowledge, there is no documented case report or study which featured acute proptosis as a clinical sign in a patient with venous hemorrhagic infarct or where acute proptosis was associated with thrombosis of a cerebral venous sinus other than cavernous sinus. This study shows that acute proptosis can be a presenting sign even in venous hemorrhagic infarct and acute proptosis can be associated with cerebral sinus venous thrombosis even without the involvement of cavernous sinus. So although rare, venous hemorrhagic infarct and cerebral venous sinus thrombosis irrespective of the venous sinus involved should be considered in any patient presenting to the emergency department with acute onset proptosis.

## Introduction

Proptosis, also known as exophthalmos, is the protrusion of the eyeball from the orbit [1]. Acute proptosis occurs within several minutes, hours, or up to two days. Subacute proptosis is proptosis occurring over weeks, and chronic proptosis is proptosis occurring over several months [2]. Studies and case reports on acute proptosis are rare and are mainly based on trauma patients and patients with carotid cavernous fistula [3]. It is a very rarely seen presenting sign in the emergency department. There are isolated case reports of patients with trauma patients with extradural hemorrhage and acute proptosis with retrobulbar hemorrhage [4,5].

Case reports of acute proptosis are very few. Thus each new case is essential. This case report is of a 38-year-old lady who presented to our emergency department with a headache for three days, altered sensorium for eight hours, and acute proptosis for 40 minutes. She was diagnosed with a venous hemorrhagic infarct in the left parietal-temporal-occipital region with thrombosis of left transverse and sigmoid sinuses, mass effect in the form of midline shift to the right side, an intraventricular extension of hemorrhage onto the left lateral ventricle.

To the best of our knowledge, no documented case report or study featured acute proptosis as a clinical sign in a patient with venous hemorrhagic infarct or where acute proptosis was associated with thrombosis of a cerebral venous sinus other than cavernous sinus. This case report shows that acute proptosis can be a presenting sign even in venous hemorrhagic infarct, and acute proptosis can be associated with cerebral sinus venous thrombosis even without the involvement of the cavernous sinus.

## Case presentation

A 38-year-old lady presented to the emergency department with a headache for three days, altered sensorium for eight hours, and proptosis of the left eye for 40 minutes. The headache started around three days back. It was gradual in onset, persistent with medium severity. The patient was taken to a local clinic, and medicines were prescribed, but the headache persisted. Around eight hours back, at around noon, she developed altered sensorium and multiple episodes of vomiting. She was taken to a local hospital where a computerized tomography (CT) scan of the brain was taken which was normal. Then she was referred to our hospital, which is a tertiary care center. According to the relatives, around 40 minutes back, the patient developed sudden proptosis of the left eye, which is progressing.

On physical examination, vitals were as follows: pulse rate: 82 per minute, blood pressure: 140/80 mmHg, respiratory rate: 20 per minute, temperature: 97.5°F, and oxygen saturation: 99% on room air. On neurological examination, her Glasgow coma scale (GCS) score was 11. She opened her eyes to voice (eye response score - three); she was speaking inappropriate discernible words to pain stimulus (verbal response score - three); and she was localizing to pain with her left hand (motor response score - five). Her right pupil was 3 mm reactive to light, and her left pupil was 5 mm nonreactive to light. She was moving all her limbs, but there was right-sided weakness compared to the left. Exact power could not be assessed due to altered sensorium. Babinski’s sign was positive on the right side. On auscultation, her lungs were clear and had equal air entry bilaterally. Her heart rate was regular with normal S1 and S2, and there were no murmurs, rubs, or gallops. The abdomen was soft, non-tender, and non-distended, with normal bowel sounds.

**Figure 1:**
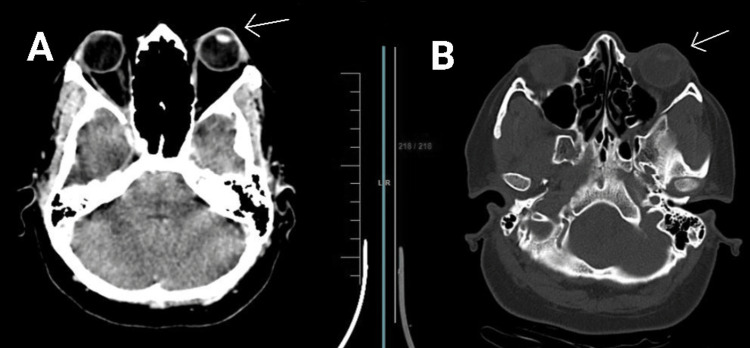
CT brain scan of the patient shows the proptosis of the left eye. (A) Brain window. (B) Bone window. Arrows indicate proptosis.

**Figure 2:**
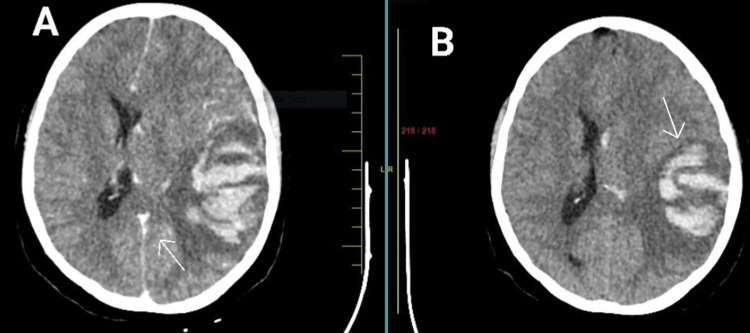
(A) CT brain scan with contrast; arrow indicates the sigmoid sinus thrombosis. (B) Non-contrast CT brain scan; arrow indicates the venous hemorrhagic infarct. Ventricular bleed and midline shift also seen.

A CT brain scan with contrast study was taken, which reported venous hemorrhagic infarct in the left parietal-temporal-occipital region with thrombosis of left transverse and sigmoid sinuses, mass effect in the form of midline shift to the right side, an intraventricular extension of hemorrhage onto the left lateral ventricle shown in Figures 1-2.

The patient was treated in the emergency department with prophylactic antiepileptics, antacids, low-molecular-weight heparin, mannitol, dexamethasone, and intravenous (IV) fluid normal saline. The patient was advised to get admitted to the neurosurgery department. However, due to financial reasons, they requested a referral to a government hospital and were referred, and the patient was lost to follow-up.

Past history: no history of trauma, surgery, oral or IV drug use, recreational drug usage, immunosuppression, hypertension, coronary artery disease, cerebrovascular accident, cancer, coagulopathy, or use of anticoagulants.

There was no family history of cerebrovascular accident, hypertension, diabetes mellitus, or coronary artery disease. The patient was a non-alcoholic, non-smoker.

## Discussion

Acute proptosis is a rare condition presenting to the emergency department. It is mainly seen in trauma patients with retrobulbar hemorrhage. There are very few case reports of patients with acute proptosis associated with cortico-cavernous fistula, trauma patients with extradural hemorrhage, retrobulbar hemorrhage, subperiosteal hemorrhage, isolated rectus muscle hematoma, intracerebral hemorrhage following thrombolysis, fever with orbital cellulitis and subperiosteal hemorrhage that have been reported [3-9]. Thus each new case is essential.

This case report is of a 38-year-old lady who presented to our emergency department with a headache for three days, altered sensorium for eight hours, and acute proptosis for 40 minutes. She was diagnosed with a venous hemorrhagic infarct in the left parieto-occipitotemporal region with thrombosis of the left transverse and sigmoid sinuses. Following an extensive search, we could not find a documented case report or study which featured acute proptosis as a clinical sign in a patient with venous hemorrhagic infarct or where acute proptosis was associated with thrombosis of a cerebral venous sinus other than cavernous sinus. This study shows that acute proptosis can be a presenting sign even in venous hemorrhagic infarct, and acute proptosis can be associated with cerebral sinus venous thrombosis without cavernous sinus involvement.

Studies and case reports of acute proptosis are very few. Most case reports are of patients with retrobulbar hemorrhage presenting to the emergency department following trauma and those with carotico-cavernous fistula. According to some case reports, acute proptosis may also occur in patients with cavernous sinus thrombosis and trauma patients with extradural hemorrhage, retrobulbar hemorrhage, subperiosteal hemorrhage, and isolated rectus muscle hematoma. There are also isolated case reports of patients with acute proptosis with retrobulbar hemorrhage and intracerebral hemorrhage following thrombolysis and fever with orbital cellulitis and subperiosteal hemorrhage [3-9]. According to one detailed study, there was no association found between a specific type of cerebral venous sinus and ocular signs other than proptosis. Proptosis was the only ocular sign associated with a specific cerebral sinus, the cavernous sinus. The absence of proptosis and vision loss was seen to have a good prognosis in patients with cavernous venous thrombosis [10].

Cerebral venous infarction is a rare type of stroke. It is most commonly caused by the thrombosis of the cerebral venous sinuses or the cerebral veins. When the infarct occurs in an uncommon area, cerebral venous infarction should be considered among the differential diagnoses. Cerebral venous infarction is also commonly associated with venous hemorrhage in about half of the patients with cerebral venous infarction, and this association has a poorer prognosis [11]. The incidence of cortical venous sinus thrombosis is approximately two-five per million, which is the reason for about 1% of total strokes. Females and the young, middle-aged population are more affected [11]. Thrombosis of the cortical venous sinuses commonly affects more than one sinus. The most commonly affected sinus is the left transverse sinus. The right transverse, superior sagittal jugular, deep cerebral veins including straight and Galen, and cavernous sinus are the next most commonly affected in the decreasing order of incidence [11].

The risk factors of thrombosis are hypercoagulability, injury to the vessel wall, and alterations in blood flow, which are together described as Virchow’s triad. Deficiency of protein C, protein S or antithrombin, pregnancy, puerperium, and use of estrogen-containing contraceptive pills are conditions leading to hypercoagulability. Chronic inflammatory diseases like inflammatory bowel diseases, lupus, Behcet’s disease, etc., are conditions where the vessel wall is affected. These conditions are associated with cortical venous sinus thrombosis. Hematological disorders like polycythemia vera, paroxysmal nocturnal hemoglobinuria, infections and medical procedures involving ear, nose, throat, paranasal sinuses, sickle cell anemias, homocysteinemia, dehydration, COVID-19, iron deficiency anemia in children, malignancies are other conditions associated with cortical venous sinus thrombosis [12].

The most common symptom associated with cortical venous sinus thrombosis is a headache which can vary in severity from mild to severe. Other commonly seen symptoms are motor weakness due to infarction, ocular signs and symptoms, seizures, and altered sensorium. Concerning ocular findings in cortical venous sinus thrombosis, papilledema is the most common. Sixth nerve palsy, diplopia, vision loss, and proptosis are other ocular signs and symptoms associated with cortical venous sinus thrombosis. As cortical venous sinus thrombosis is commonly associated with infarction and hemorrhage and has a poor prognosis, it has to be rapidly diagnosed, and treatment should be started rapidly. A CT scan or a magnetic resonance imaging (MRI) scan of the brain can diagnose cortical venous sinus thrombosis. Anticoagulation should be started as soon as cortical venous sinus thrombosis is diagnosed. It is important to note that venous hemorrhage associated with cortical venous sinus thrombosis is not a contraindication for anticoagulation therapy [13,14].

## Conclusions

Acute proptosis is usually seen in trauma with retrobulbar hemorrhage and cortico-cavernous fistula, but in the case of our patient who presented to the emergency department, it was a venous hemorrhagic infarct. This was a dilemma of diagnosis. So clinically along with other differential diagnoses, venous hemorrhagic infarct should also be ruled out. Also, it is uncommon for proptosis to be associated with cortical venous sinuses other than cavernous sinuses according to studies. Our patient had thrombosis of the left transverse and sigmoid sinuses, which shows that proptosis can occur acutely even without the cavernous sinus being involved. Though the case report has no treatment details or follow-up, this case adds to the limited medical literature on acute onset proptosis.
